# Causal analysis of case-control data

**DOI:** 10.1186/1742-5573-3-2

**Published:** 2006-01-27

**Authors:** Stephen C Newman

**Affiliations:** 1 Department of Psychiatry, Mackenzie Health Sciences Centre, University of Alberta, Edmonton, Alberta, T6G 2B7, Canada

## Abstract

In a series of papers, Robins and colleagues describe inverse probability of treatment weighted (IPTW) estimation in marginal structural models (MSMs), a method of causal analysis of longitudinal data based on counterfactual principles. This family of statistical techniques is similar in concept to weighting of survey data, except that the weights are estimated using study data rather than defined so as to reflect sampling design and post-stratification to an external population. Several decades ago Miettinen described an elementary method of causal analysis of case-control data based on indirect standardization. In this paper we extend the Miettinen approach using ideas closely related to IPTW estimation in MSMs. The technique is illustrated using data from a case-control study of oral contraceptives and myocardial infarction.

## Introduction

In a series of papers, Robins and colleagues describe inverse probability of treatment weighted (IPTW) estimation in marginal structural models (MSMs) [1-7], a method of causal analysis of longitudinal data based on counterfactual principles. This family of statistical techniques is similar in concept to weighting of survey data, except that weights are estimated using study data rather than defined so as to reflect sampling design and post-stratification to an external population. Several decades ago Miettinen [8] described an elementary method of causal analysis of case-control data based on indirect standardization. In this paper we extend the Miettinen approach using ideas closely related to IPTW estimation in MSMs. For simplicity we ignore random error until the illustrative example.

### Population-based incidence case-control study

Consider a population-based case-control study having an incidence design, that is, one in which only incident cases are eligible for recruitment. Let *E *be a dichotomous variable (0: absent, 1: present) representing the exposure of interest, and let *F *be a polychotomous variable (*i *= 0,1, ..., *I*), which we later treat as a confounder. At any time point we may think of the population as being comprised of exposed and unexposed (sub)populations. Suppose that recruitment of cases and controls takes place over a period of *T *years. We assume that during the period of recruitment the exposed and unexposed populations are stationary (i.e., independent of time) with respect to population size and incidence rate (of disease) in each of the strata of *F *[9]. Provided that *T *is not too large, say no more than two or three years, this assumption is likely to be approximately satisfied in practice.

Let *N*_1*i *_be the number of people in the *i*th stratum of the exposed population who are free of disease (at any time during the period of recruitment), and let *N*_0*i *_be the corresponding number in the *ith *stratum of the unexposed population. Let  and . Therefore at any time during the period of recruitment, there are *N*_1 _exposed and *N*_0 _unexposed people in the population "at risk" of disease, hence eligible to be controls. Since the population is stationary, we may assume that controls are selected at the end of the period of recruitment. This avoids the inconvenience of having a control selected early in the study become a case later on. In practice, controls are usually sampled throughout the period of recruitment, with one or more controls enrolled as each case enters the study. The case triggering this activity and the associated controls can be thought of as a matched set, where the matching variable is "time." This method of subject recruitment is a type of risk set sampling and, in theory, should be followed by a conditional statistical analysis [10]. Generally, matching on time is ignored in the analysis of case-control data, which in practical terms is not that different from making the stationary population assumption.

Let *R*_1*i *_and *R*_0*i *_be the incidence rates (of disease) in the *i*th stratum of the exposed and unexposed populations, respectively. The crude incidence rates are



and



The impact of exposure can be measured using the standardized morbidity ratio, which has different forms depending on the choice of standard population [11]. Taking the standard population to be, in turn, the exposed, unexposed, and total (exposed plus unexposed) populations, the corresponding standardized morbidity ratios are



and



We now view the population as an open (dynamic) cohort that is followed over the period of recruitment, with onset of disease as the endpoint of interest [12]. Entry into the cohort occurs, for example, as a result of birth and in-migration, and censoring takes place when, for instance, there is out-migration and death from a cause other than the disease of interest.

### Simple random sampling

Assume that cases and controls are sampled using simple random sampling. Let γ and λ be the sampling probabilities for cases and controls, respectively; that is, γ is the proportion of eligible cases enrolled in the study during the period of recruitment, and λ is the corresponding proportion of controls. We assume that these are also the sampling probabilities within each of the strata of *E × F*, the cross-classification of *E *and *F*. It follows from the stationary population assumption that over the period of recruitment the number of person-years experienced by individuals in the *i*th stratum who are exposed and at risk of disease is *N*_1*i*_*T*. The corresponding number of (incident) cases is *R*_1*i*_*N*_1*i*_*T*, with *a*_1*i *_= γ*R*_1*i*_*N*_1*i*_*T *of them recruited into the study. Likewise, the number of cases recruited into the study among individuals in the *i*th stratum who are unexposed and at risk of disease is *a*_0*i *_= γ*R*_0*i*_*N*_0*i*_*T*. In view of remarks made above, *b*_1*i *_= λ*N*_1*i *_exposed and *b*_0*i *_= λ*N*_0*i *_unexposed controls will be recruited into the study from the *ith *stratum. Table 1 summarizes these observations.

**Table 1:** Number of cases and controls in *i*th stratum of *F *under simple random sampling

E	Case	Control
1	*a*_1*i *_= γ*R*_1*i*_*N*_1*i*_*T*	*b*_1*i *_= λ*N*_1*i*_
0	*a*_0*i *_= γ*R*_0*i*_*N*_0*i*_*T*	*b*_0*i *_= λ*N*_0*i*_

It follows from Table 1 that



and



which shows that *SMR*_E_, *SMR*_U _and *SMR*_T _can be estimated from incidence case-control data [13-15]. Note that nowhere have we made the rare disease assumption.

We are interested in measuring the causal effect of exposure on the exposed cohort using counterfactual methods [16-21]. To accomplish this we imagine the group of individuals in the exposed cohort *prior to exposure *and consider two scenarios: in the first, exposure subsequently occurs (as it does in reality); in the second, exposure does not occur. The second scenario is counterfactual because it rests on the hypothetical condition that exposure does not take place, when in fact it does. By contrasting outcomes arising out of the two scenarios we are able to define parameters having a causal interpretation. This is because we are (in theory) comparing two groups of individuals that are identical except for exposure status. The crude incidence rate corresponding to the first scenario is *R*_1_. Denote the crude incidence rate for the second scenario by *R*_1_^*^. Even though the second scenario is counterfactual, it is possible, provided certain assumptions are satisfied, to estimate *R*_1_^*^, as discussed below.

In practice, the unexposed cohort, not the exposed cohort under the counterfactual condition, is used for comparative purposes. To the extent that the two associated incidence rates, *R*_0 _and *R*_1_^*^, differ, we say that there is confounding. More precisely, the counterfactual definition of confounding states that confounding is present if and only if *R*_0 _≠  *R*_1_^*^[16-21].

We now make two fundamental assumptions: (1) *E *does not "affect" *F *(in particular, *F *is not on a causal pathway between *E *and the disease), and (2) there is no confounding (according to the counterfactual definition) in the strata of *F*. Using arguments analogous to those in [21] and [22], we have



Since there is no confounding in the strata of *F*, when *confounding *is present, that is, *R*_0 _≠ *R*_1_^*^, we attribute it to *F *and say that *F *is a *confounder*. It follows from (1), (2) and (4) that



which shows that under the above two assumptions, *SMR*_E _has a causal interpretation.

Following the approach of Sato and Matsuyama [11], we assign each exposed subject in the *i*th stratum the weight 1, and each unexposed subject the weight *b*_1*i*_/*b*_0*i*_. We refer to these weights as the empirical weights. Note that *b*_1*i*_/*b*_0*i *_is the odds that a control in *ith *stratum is exposed. From Table 2, which gives case-control counts after applying these weights, we see that *SMR*_E _can be interpreted as a weighted odds ratio. Accordingly, in the case-control setting we denote *SMR*_E _by s*OR *and refer to it as the standardized odds ratio.

**Table 2:** Weighted number of cases and controls under simple random sampling

E	Case	Control
1		
0		

Let



and *n*_*i *_= *a*_1*i *_+ *a*_0*i *_+ *b*_1*i *_+ *b*_0*i*_. It is readily demonstrated that s*OR *as given by (3) and the Mantel-Haenszel odds ratio estimate *OR*_MH _[23] can be expressed as weighted sums of the *OR_i_*:



These expressions differ only to the extent that the relative magnitudes of the *b*_0*i *_and *n*_*i *_vary across strata. For case-control studies in which unexposed controls constitute the majority of subjects, s*OR *and *OR*_MH _will be close in value.

It was pointed out by Greenland [15] that *OR*_MH _does not have an epidemiologic interpretation when there is effect modification. This is because the stratum-specific weights in (6) do not reflect a recognizable target population. With s*OR *the target population is clearly specified (namely, the exposed population), and so s*OR *has a causal interpretation even in the presence of effect modification. This is advantageous in a number of settings. Consider the familiar situation in which, after stratification by one or more confounders, the stratum-specific odds ratio estimates do not exhibit a meaningful pattern, or the differences in these estimates can be distinguished on statistical grounds but are of no practical importance. When this occurs it is desirable to have recourse to a summary odds ratio estimate, even though effect modification may be present.

### Stratified random sampling

Let *G *be a polychotomous variable (*j = *0, 1, ..., *J*) and suppose that cases and controls are sampled using stratified random sampling based on the strata of *G*. Let γ_*j *_and λ_*j *_be the sampling probabilities for cases and controls in the *j*th stratum, respectively. We assume that these are also the sampling probabilities for the exposed and unexposed populations in the *j*th stratum. Corresponding to Tables 1 and 2 we have Tables 3 and 4, from which it follows that

**Table 3:** Number of cases and controls in *ij*th stratum of *F × G *under stratified random sampling

E	Case	Control
1	*a*_1*ij *_= γ_*j*_*R*_1*ij*_*N*_1*ij*_*T*	*b*_1*ij *_= λ_*j*_*N*_1*ij*_
0	*a*_0*ij *_= γ_*j*_*R*_0*ij*_*N*_0*ij*_*T*	*b*_0*ij *_= λ_*j*_*N*_0*ij*_

**Table 4:** Weighted number of cases and controls under stratified random sampling

E	Case	Control
1		
0		



Under stratified random sampling, we assign each exposed subject in the *ij*th stratum the (empirical) weight 1/γ_*j*_, and each unexposed subject the weight *b*_1*ij*_/γ_*j*_*b*_0*ij*_. As before, in the case-control context we denote *SMR*_E _by s*OR.*

### MSM-IPTW approach

When there are multiple confounders, the data can be stratified according to their cross-classification and the above method used. However, this may lead to cells with small or zero entries, resulting in instability of estimates. A statistically more efficient alternative is to adopt the MSM-IPTW approach and obtain the weights (for controls) from a logistic regression analysis of control data, where *E *is the dependent variable and the confounders (of the *E-*disease association) are the independent variables. We refer to these weights as regression weights.

Under simple random sampling, the weight for each exposed subject is set equal to 1, and the weight for each unexposed subject is taken to be the fitted odds for that individual. For stratified random sampling, the logistic regression analysis of control data must include the stratifying variable. In the *j*th stratum, the weight for each exposed subject is set equal to the reciprocal of the sampling probability, and the weight for each unexposed subject is taken to be the fitted odds for that individual multiplied by the reciprocal of the sampling probability.

Once the regression weights have been calculated, the odds ratio for the exposure-disease association is estimated from a weighted logistic regression analysis using generalized estimating equations (GEE) [24], where *E *is the sole independent variable. As remarked by Hernán et al. [6], it has been shown by Robins [1,2] that for longitudinal data where there are no unmeasured confounders and where a certain positivity assumption is met, the weighted GEE approach produces an asymptotically unbiased estimate of the causal parameter. Depending on the software used for the GEE analysis, it may be necessary to scale the weights such that their sum across all cases equals the actual number of cases, and likewise for controls.

### Example

Table 5 presents data from an incidence case-control study of oral contraceptives (OC) and myocardial infarction (MI) [25]. We are interested in measuring the causal effect of oral contraceptive use on myocardial infarction in women taking this medication; that is, the target population is women taking oral contraceptives. For the purposes of illustration, we assume that age (AGE) and cigarettes (CIG) are sufficient to control confounding and that there is no misclassification or other source of bias.

**Table 5:** Case-control study of oral contraceptives and myocardial infarction [25]

CIG		AGE						Total	
				
		25–34		35–44		45+			
	OC	Case	Control	Case	Control	Case	Control	Case	Control
none	1	0	38	1	12	3	2	4	52
	0	1	281	13	318	20	155	34	754
		= 2.44		= 2.03		= 11.63		= 1.71	
									
1–24	1	2	35	1	15	0	1	3	51
	0	5	221	32	249	42	96	79	566
		= 2.53		= 0.52		= 0.76		= 0.42	
									
25+	1	11	22	8	8	3	2	22	32
	0	8	112	53	125	31	50	92	287
		= 7.00		= 2.36		= 2.42		= 2.14	

Total	1	13	95	10	35	6	5	29	135
	0	14	614	98	692	93	301	205	1607
		= 6.00		= 2.02		= 3.88		= 1.68	

OC: oral contraceptives

CIG: cigarettes

AGE: age

We first performed a standard logistic regression analysis, with MI as the dependent variable and OC, AGE and CIG as the independent variables. As pointed out by Greenland and Maldonado [26], there are problems identifying the target population when using standard logistic regression analysis. Models were fit using EGRET [27]: statistical significance of individual terms was determined using the likelihood ratio test, and the goodness-of-fit statistic *G*^2 ^was based on the deviance. On purely statistical grounds the best-fitting model had main effects for OC, AGE and CIG, along with the interaction term AGE × CIG (*G*^2 ^= 12.0, df = 8, *p = *.15). The odds ratio estimate for the OC-MI association was 2.82 (95% confidence interval [CI]: 1.70,4.68). Of note, the Mantel-Haenszel odds ratio estimate, *OR*_MH_*= *2.82 (95% CI: 1.70,4.69), was virtually identical to the logistic regression estimate. The *OR*_MH _confidence interval was based on the variance estimate described by Robins, Breslow and Greenland [28,29]. The model with main effects for OC, AGE and CIG, along with the interaction term OC × CIG also fit the data quite well (*G*^2 ^= 17.4, df = 10, *p = *.068). Given that oral contraceptive use is the exposure of interest, it is reasonable – on substantive grounds – to consider this as the "final" model. If so, because of the OC × CIG interaction, the model no longer provides a summary estimate of the odds ratio for the OC-MI association.

Next, we conducted an analysis using the MSM-IPTW approach. To obtain regression weights, a standard logistic regression analysis of control data was performed, with OC as the dependent variable, and with AGE and CIG as the independent variables. The best-fitting model had only a main effect for AGE (*G*^2^*= *5.06, df = 6, *p = *.54). We then conducted a weighted logistic regression analysis using generalized estimating equations, with MI as the dependent variable and OC as the sole independent variable. Following Hernán et al. [4] and Sato and Matsuyama [11], calculations were performed using the SAS procedure PROC GENMOD [30]. The odds ratio estimate for the OC-MI association was 3.34 (95% CI: 2.15, 5.21). Interestingly, when empirical weights were used instead of regression weights, the odds ratio estimate (which equals s*OR*) was 2.83 (95% CI: 1.82,4.41). This is very close to the odds ratio and confidence interval estimates based on the standard logistic regression and Mantel-Haenszel analyses.

## Discussion

The counterfactual definition of confounding represents an important conceptual advance over earlier formulations of confounding. Working within the counterfactual framework, Robins and colleagues developed inverse probability of treatment weighted estimation in marginal structural models for the analysis of longitudinal data [1-7]. Although primarily aimed at the problem of time-dependent confounding, this method is valid when confounders are independent of time.

Extending the work of Miettinen [8], in this paper we present a method of causal analysis of case-control data that is closely related to IPTW estimation in MSMs. We consider only case-control studies conducted in a stationary population. Provided the time period during which the study is conducted is not too long, it may be reasonable to regard the population as at least approximately stationary. Whether strictly valid or not, the stationary population assumption appears to be made routinely – usually implicitly – when case-control studies are conducted. An alternative is to match controls to cases on time of recruitment using risk set sampling [10] and perform a conditional data analysis. Under the rare disease assumption, approximate parameter estimates can then be obtained using the MSM-IPTW approach [7].

## Declaration of competing interests

The author(s) declare that they have no competing interests.
